# Efficacy and safety of abobotulinumtoxinA for upper limb spasticity in children with cerebral palsy: a randomized repeat‐treatment study

**DOI:** 10.1111/dmcn.14733

**Published:** 2020-11-18

**Authors:** Mauricio R Delgado, Ann Tilton, Jorge Carranza‐Del Río, Nigar Dursun, Marcin Bonikowski, Resa Aydin, Iwona Maciag‐Tymecka, Joyce Oleszek, Edward Dabrowski, Anne‐Sophie Grandoulier, Philippe Picaut, the Dysport in PUL study group, Anne Renders, Josef Kraus, Eduard Minks, Uri Givon, Yair Sadaka, Daniel Weigl, Aviva Fattal‐Valevski, Hilla Ben‐Pazi, Jose Alberto Moreno Gonzalez, Elsa Maria Ivon Perez Flores, Marek Jozwiak, Roser Garreta Figuera, Xenia Alonso Curco, Mar Melendez Plumed, Ozlen Peker, John P Phillips, Gadi Revivo, Sarah H Evans, Edward A Wright, Jenny Lupovici Wilson, Heakyung Kim, Shawn Aylward, Mark E Gormley

**Affiliations:** ^1^ Department of Neurology University of Texas Southwestern Medical Center Scottish Rite Hospital for Children Dallas TX USA; ^2^ Department of Neurology LSUHSC and Children's Hospital New Orleans New Orleans LA USA; ^3^ Hospital San José Celaya Celaya, Guanajuato Mexico; ^4^ Department of Physical Medicine and Rehabilitation Faculty of Medicine Kocaeli University Kocaeli Turkey; ^5^ Mazovian Neuropsychiatry Center Zagórze, nr Warsaw Poland; ^6^ Department of Physical Medicine and Rehabilitation Istanbul Faculty of Medicine Istanbul University Istanbul Turkey; ^7^ Rehabilitation Center KROK PO KROKU Gdansk Poland; ^8^ Department of Physical Medicine and Rehabilitation University of Colorado and Children's Hospital Colorado Aurora CO USA; ^9^ Department of Pediatric Physical Medicine and Rehabilitation Beaumont Health Oakland University School of Medicine Grosse Pointe MI USA; ^10^ Atlanstat consultant for Ipsen Pharma Les Ulis France; ^11^ Ipsen Pharma Cambridge MA USA

## Abstract

**Aim:**

To assess the efficacy and safety of repeat abobotulinumtoxinA injections in reducing upper limb spasticity in children with cerebral palsy (CP).

**Method:**

This was a double‐blind, repeat‐cycle study (NCT02106351) in children with CP (2–17y). Children were randomized to receive 2U/kg (control), 8U/kg, or 16U/kg abobotulinumtoxinA injections into the target muscle group (wrist or elbow flexors) and additional muscles alongside occupational therapy via a home‐exercise therapy program (HETP; minimum five 15min sessions/wk). Children received 8U/kg or 16U/kg plus HETP in cycles 2 to 4.

**Results:**

During cycle 1, 210 children (126 males, 84 females; mean age [SD] 9y [4y 5mo], range 2–17y; *n*=70/group) had at least one upper limb abobotulinumtoxinA injection and 209 complied with the HETP. At week 6 of cycle 1, children in the 8U/kg or 16U/kg groups had significantly lower Modified Ashworth scale scores versus the 2U/kg group (primary outcome: treatment differences of –0.4 [*p*=0.012] and –0.7 [*p*<0.001] respectively). All groups improved on Physician Global Assessment and children in all groups achieved their treatment goals at least as expected. Therapeutic benefits were sustained during cycles 2 to 4; muscular weakness was the only treatment‐related adverse event reported in at least one child/group (4.3% and 5.7% vs 1.4% respectively).

**Interpretation:**

Treatment with 8U/kg or 16U/kg abobotulinumtoxinA significantly reduced upper limb spasticity versus the 2U/kg control dose. Therapeutic benefits of abobotulinumtoxinA plus HETP were sustained with repeat treatment cycles.

**What this paper adds:**

- AbobotulinumtoxinA injections significantly reduced upper limb spasticity in children with cerebral palsy.
- Children treated with abobotulinumtoxinA and targeted home exercises showed global improvement and goal attainment.
- Benefits were sustained over 1 year with repeat cycles of abobotulinumtoxinA and home exercises.
- AbobotulinumtoxinA injections into the upper limb were well tolerated over 1 year.

## What this paper adds

- AbobotulinumtoxinA injections significantly reduced upper limb spasticity in children with cerebral palsy.
- Children treated with abobotulinumtoxinA and targeted home exercises showed global improvement and goal attainment.
- Benefits were sustained over 1 year with repeat cycles of abobotulinumtoxinA and home exercises.
- AbobotulinumtoxinA injections into the upper limb were well tolerated over 1 year.

This article is commented on by Graham on page 499 of this issue.

This article's abstract has been translated into Spanish and Portuguese.

Follow the links from the abstract to view the translations.

## Eficacia y seguridad de abobotulinumtoxinA para la espasticidad de las extremidades superiores en niños con parálisis cerebral: un estudio aleatorizado de repetición del tratamiento

**Objetivo:**

Evaluar la eficacia y seguridad de las inyecciones repetidas de abobotulinumtoxinA para reducir la espasticidad de las extremidades superiores en niños con parálisis cerebral (PC).

**Método:**

Este fue un estudio doble ciego de ciclo repetido (NCT02106351) en niños con PC (2‐17 años). Los niños fueron aleatorizados para recibir inyecciones de abobotulinumtoxinA de 2U / kg (control), 8U / kg o 16U / kg en el grupo de músculos objetivo (flexores de la muñeca o del codo) y músculos adicionales junto con la terapia ocupacional a través de un programa de terapia de ejercicio en el hogar (HETP; mínimo cinco sesiones de 15min / semana). Los niños recibieron 8U / kg o 16U / kg más HETP en los ciclos 2 a 4.

**Resultados:**

Durante el ciclo 1, 210 niños (126 varones, 84 mujeres; edad media [DE] 9 años [4 años 5 meses], rango 2‐17 años) (n = 70 / grupo) recibieron al menos una inyección de abobotulinumtoxinA en el miembro superior y 209 cumplieron con la HETP. En la semana 6 del ciclo 1, los niños de los grupos de 8U / kg o 16U / kg tenían puntuaciones de la escala de Ashworth modificadas significativamente más bajas en comparación con el grupo de 2U / kg (resultado primario: diferencias de tratamiento de –0,4 [p = 0,012] y –0,7 [p <0,001] respectivamente). Todos los grupos mejoraron en la escala Evaluación Global del Médico, y los niños de todos los grupos lograron sus objetivos de tratamiento al menos como se esperaba. Los beneficios terapéuticos se mantuvieron durante los ciclos 2 a 4; la debilidad muscular fue el único evento adverso relacionado con el tratamiento informado en al menos un niño / grupo (4,3% y 5,7% frente a 1,4% respectivamente).

**Interpretación:**

El tratamiento con 8U/kg o 16U/kg de abobotulinumtoxinA redujo significativamente la espasticidad de las extremidades superiores en comparación con la dosis de control de 2U/kg. Los beneficios terapéuticos de abobotulinumtoxinA más HETP se mantuvieron con ciclos de tratamiento repetidos.

## Eficácia e segurança da abobotulinumtoxina A para espasticidade do membro superior em crianças com paralisia cerebral: um estudo randomizado de tratamento repetido

**Objetivo:**

Avaliar a eficácia e segurança de injeções repetidas de abobotulinumtoxina A na redução da espasticidade do membro superior em crianças com paralisia cerebral (PC).

**Método:**

Este foi um estudo duplo cego, com ciclo repetido (NCT02106351) em crianças com PC (2–17a). As crianças foram randomizadas para receber injeções de 2U/kg (controle), 8U/kg, ou 16U/kg de abobotulinumtoxina A no grupo muscular alvo (flexores de punho ou cotovelo) e músculos adicionais junto com um programa de terapia ocupacional com exercícios domiciliares (PTOED; mínimo de 5 sessões/semana de 15 minutos). As crianças receberam 8U/kg ou 16U/kg mais PTOED em ciclos de 2 a 4.

**Resultados:**

Durante o ciclo 1, 210 crianças (126 do sexo masculino, 84 do sexo feminino; media de idade [DP] 9a [4a 5m], variação 2–17a) (*n*=70/grupo) tiveram ao menos uma injeção de abobotulinumtoxina A no membro superior e 209 seguiram o PTOED. Na semana 6 do ciclo1, crianças nos grupos 8U/kg ou 16U/kg tiveram escores da escala Ashworth significativamente menores versus o grupo 2U/kg (desfecho primário: diferenças no tratamento de –0,4 [*p*=0,012] e –0,7 [*p*<0,001] respectivamente). Todos os grupos melhoraram na Avaliação Médica Global e crianças de todos os grupos atingiram os objetivos de tratamento pelo menos como esperado. Benefícios terapêuticos foram mantidos durante os ciclos 2 a 4; fraqueza muscular foi o único evento adverso relacionado ao treinamento em pelo menos uma criança/ grupo (4,3% e 5,7% vs 1,4% respectivamente).

**Interpretação:**

O tratamento com 8U/kg ou 16U/kg de abobotulinumtoxina reduziu significativamente a espasticidade do membro superior versus a dose controle de 2U/kg. Benefícios terapêuticos da abobotulinumtoxina A mais PTOED foram mantidos com os repetidos ciclos de tratamento.

**Abbreviations**
AHA: Assisting Hand Assessment
GAS: Goal attainment scaling
HETP: Home‐exercise therapy program
MAS: Modified Ashworth scale
MTS: Modified Tardieu scale
PGA: Physician Global Assessment
PTMG: Primary targeted muscle group
TEAE: Treatment‐emergent adverse event

Upper limb impairment is common in individuals with cerebral palsy (CP) and is an important source of disability.^1^, ^2^ Combinations of spasticity, poor selective motor control, weakness, sensory impairment, dystonia, decreased range of motion, and other deficits contribute to difficulties in reaching, grasping, releasing, and manipulating objects.^3^, ^4^ Considering the profound impact of upper limb impairment on daily life, treatment teams often commit considerable time and resources towards rehabilitation.

The cornerstone of treatment is occupational therapy and/or physiotherapy, which is often combined with antispasticity pharmacotherapy in a long‐term, multidisciplinary rehabilitation program.^5^ Since its introduction in the 1990s,^6^ botulinum neurotoxin A (BoNT‐A) has become an established treatment for focal spasticity in children with CP.^7^, ^8^ While already included in current national and international guidelines,^8^, ^9^, ^10^ until recently, the available evidence for BoNT‐A was not sufficient for regulatory authority approval in pediatric upper limb spasticity and an appropriately designed study was required to enable children to have full ‘on‐label’ access to this treatment at established doses.

AbobotulinumtoxinA (Dysport^®^; Ipsen Biopharm, Wrexham, UK) is a formulation of BoNT‐A proven to reduce spasticity and improve function in children with lower‐limb impairment due to CP.^11^ The primary aim of this phase 3 study was to confirm the efficacy and safety of repeat abobotulinumtoxinA injections in combination with a personalized, goal‐oriented home‐exercise therapy program (HETP)^12^ in reducing spasticity in children with CP. It is the first double‐blind, randomized study to compare two doses of abobotulinumtoxinA (8U/kg and 16U/kg) versus a low‐dose (2U/kg) active control group. Secondary and exploratory objectives of the study were to evaluate the impact of treatment (i.e. abobotulinumtoxinA plus HETP) on overall upper limb function.

## Method

### Study design and participants

This was a double‐blind, randomized, repeat treatment (up to four cycles over 1y), phase 3 study conducted at 32 sites across Belgium, the Czech Republic, Poland, Spain, Turkey, Israel, Mexico, and the USA. The study began on 10th April 2014 and was completed on 4th September 2018. Investigators at each center received training and certification for the Modified Ashworth scale (MAS),^13^ Physician Global Assessment (PGA) of treatment response, Modified Tardieu scale (MTS),^14^ and goal attainment scaling (GAS).^15^

Children (aged 2–17y weighing ≥10kg) with a diagnosis of CP^16^ and increased muscle tone/spasticity in at least one upper limb were eligible for inclusion if they had a MAS score of at least 2 in the primary targeted muscle group (PTMG; elbow or wrist flexors). We included a broad range of disease severity, from Gross Motor Function Classification System (GMFCS) level I to IV.^17^ Any physiotherapy or occupational therapy had to have been initiated at least 30 days before the baseline visit and continued over the injection cycle. Children with a fixed contracture in the PTMG (defined as range of motion angle of <40°, regardless of the starting and finishing angles, measured at the MTS slow [X_V1_] speed at the baseline visit) were excluded from this study. The available range for wrist flexors was measured without holding the fingers and allowing free finger flexion. Other key exclusion criteria were choreoathetoid/dystonic movements, history of aspiration or dysphagia, previous/planned surgery of the PTMG, and phenol/alcohol injections within the past year. Children who had had previous BoNT‐A treatment within the 6 months before the study in the study limb or within the 3 months in other body parts were excluded, as were those treated with baclofen within 30 days before the study.

In addition to any existing physiotherapy or occupational therapy (which continued throughout the study), all children were to participate in a personalized, goal‐oriented HETP to provide a standardized background of good practice after BoNT‐A therapy.^12^ The minimum expected requirement for the HETP was five 15‐minute sessions per week and the HETP was mainly designed to support the primary treatment goal chosen. Eligibility for retreatment in the next treatment cycle was assessed at week 16; if not eligible for retreatment, they returned every 6±2 weeks until they required retreatment, or until week 52. Up to four treatments could be administered in the study.

### Ethical standards

The study was conducted in compliance with the Declaration of Helsinki, Good Clinical Practice guidelines, and with approval of all relevant institutional review boards and ethics committees for each participating site (Appendix S1, online supporting information). Written informed consent was obtained from parents or guardians, and children when applicable, before entry into the study. This study is registered with ClinicalTrials.gov (NCT02106351).

### Dosing, randomization, and blinding

For cycle 1, patients were randomized 1:1:1 to receive 2U/kg (maximum total body dose of 80U), 8U/kg (maximum total body dose of 320U), or 16U/kg (maximum total body dose of 640U) abobotulinumtoxinA into the designated study upper limb. Randomization was stratified by age (2–9y vs 10–17y) and by prior use of BoNT (naïve vs non‐naïve). Computer‐generated lists were created by a sponsor statistician independent from the study. The study doses (8U/kg and 16U/kg) were chosen in line with clinical experience.^7^ Since use of a placebo was considered unethical in this study, the control group was given a 2U/kg dose, which was assumed to be a subtherapeutic dose based on available data and clinical experience. Doses were specific to abobotulinumtoxinA.

The total dose for the upper limb was administered in a fixed total volume of 1.6mL for all dose groups; this was divided across the PTMG and other upper limb muscles that were selected based on clinical presentation, and to support the individualized treatment goals. The PTMG was always injected with a predefined volume and maximum volumes were defined for the other upper limb muscles. To maintain blinding, study medication was prepared by site personnel not involved in any other study activities. Injections were performed with electrostimulation and/or ultrasound guidance at sites usual practice for anesthesia and pain management.

For subsequent treatment cycles (cycles 2–4), the allocated dose also remained double‐blind and an injection volume of 1.6mL continued to be administered across the PTMG and other muscles. Children initially allocated to the 2U/kg low‐dose control group were randomized to receive either 8U/kg or 16U/kg abobotulinumtoxinA, while those initially randomized to the 8U/kg and 16U/kg groups were to continue to receive their allocated doses. However, dose adaptation was permitted at the investigator’s request (reduction in case of adverse events to a minimum of 2U/kg or increase for improved efficacy to a maximum 16U/kg). During cycles 2 to 4, investigators could change the PTMG and additional injections into the lower‐limbs or the other upper limb were also permitted (up to a total body dose of 30U/kg or a maximum of 1000U, whichever was lowest when both upper and lower limbs were treated).

### Outcomes

Direct pharmacological effects of abobotulinumtoxinA on muscle tone were evaluated using the MAS to assess muscle tone and the MTS to assess spasticity. The overall clinical impact of the effect on the injected muscles was assessed using the PGA of treatment response, which is a 9‐point scale rating the global response from –4 (markedly worse) to 4 (markedly improved), and was performed by a separate evaluator to the MAS. Functional impacts of treatment were assessed by GAS. Up to three goal statements were identified before each treatment and categorized according to a predefined list applicable to this population and treatment program, and rated for importance and difficulty. Full details of the PGA and GAS are given in Tables S1 and S2 (online supporting information). Function was also assessed through passive range of motion and the Assisting Hand Assessment (AHA). The AHA was only assessed in children with hemiparesis treated at certified centers at baseline and week 6 of cycle 1.

Safety assessments included treatment‐emergent adverse events (TEAEs), vital signs, electrocardiogram, blood pressure, and laboratory variables. Neutralizing antibodies to BoNT‐A were measured with the mouse protection assay (after initial screening for binding antibodies).

### Statistical analyses

A sample size of 210 randomized children (*n*=70/group) was considered sufficient for a long‐term safety database (assuming a 10% drop‐out over 1y). This sample size also provided 99% power to detect significant differences in MAS scores between groups in the primary endpoint, assuming a change from baseline of 0.5 in the 8U/kg and 16U/kg groups and 0.1 in the 2U/kg group,^18^, ^19^ an SD of 0.5, and a type I error of 5%.

Efficacy analyses separately compared the results from the 8U/kg and 16U/kg groups with the 2U/kg group, and were performed using the modified intention‐to‐treat population, which included all children who received at least one study injection and had MAS scores at baseline and week 6 of cycle 1. Safety analyses were performed for children who received at least one abobotulinumtoxinA injection. All statistical tests were performed at the two‐tailed significance level of 0.05.

The primary efficacy endpoint was the change from baseline in MAS_PTMG_ at week 6 after initial treatment. Because of the ordered, categorical nature of the MAS, non‐normality was expected and analyses were performed using an analysis of covariance on the ranked changes from baseline, including treatment group, baseline MAS_PTMG_, stratification factors, and center as fixed effects. To help interpret the results, the least squares mean rank values were back‐transformed to the original scale. For the primary endpoint of change from baseline to week 6 in MAS_PTMG_, a two‐step hierarchical testing procedure was applied to control the family‐wise type I error with the sequential testing of the 16U/kg versus 2U/kg dose, followed by 8U/kg versus 2U/kg.

Similar to the MAS, the PGA was analyzed using a ranked analysis of variance (same fixed effects in model but without baseline value) and values back‐transformed to the original scale. Other efficacy endpoints were not ranked or back‐transformed and were assessed with analysis of covariance, analysis of variance, or logistic regression, as applicable. For GAS, a T score was calculated that took into account the rated importance, difficulty, and level of achievement.^15^ We calculated the proportion of children who responded to treatment according to the following definitions: reduction in the MAS_PTMG_ of ≥1, ≥2, and ≥3, an improvement of ≥1, ≥2, and ≥3 in the PGA, and the proportion of children who reached their GAS goal (primary goal and all selected goals). All statistical analyses were performed using SAS^®^ software version 9.4 or later (SAS Institute, Cary, NC, USA). Changes in cycles 2 to 4 are presented descriptively.

## Results

### Study disposition and baseline characteristics

In total, 212 children were randomized in the study, of which 210 received at least one abobotulinumtoxinA injection and 180 completed the study. Less than 9% of children discontinued in any treatment cycle with no differences between groups in the proportion of children discontinuing the study within each cycle. Overall, 95.2% completed cycle 1 and 24.8% required four treatments during the study period (Fig. S1, online supporting information). Of the 210 treated children, all but one participated in the HETP between baseline and week 6, with 50.9% (*n*=107) performing the exercises daily and an additional 32.4% (*n*=68) performing the exercises five or six times per week.

Baseline characteristics were comparable across the three groups (Table 1); over half (56.5–57.1%) of the children were aged 2 to 9 years, most (72.5–80.0%) had hemiparesis, and most (80.3%) were ambulatory.

**Table 1:** Baseline characteristics

Parameter	2U/kg AboBoNT‐A (*n*=69)	8U/kg AboBoNT‐A (*n*=69)	16U/kg AboBoNT‐A (*n=*70)
Age, mean (SD), y:mo	8:11 (4:7)	9:0 (4:4)	9:2 (4:4)
2–9y, *n* (%)	39 (56.5)	39 (56.5)	40 (57.1)
10–17y, *n* (%)	30 (43.5)	30 (43.5)	30 (42.9)
Sex, *n* (%)			
Male	38 (55.1)	45 (65.2)	42 (60.0)
Female	31 (44.9)	24 (34.8)	28 (40.0)
Weight (kg), mean (SD)	31.48 (16.5)	32.91 (18.1)	32.68 (16.4)
Pattern of paresis, *n* (%)			
Hemiparesis	54 (78.3)	50 (72.5)	56 (80.0)
Diparesis	0	3 (4.3)	2 (2.9)
Tetraparesis	14 (20.3)	14 (20.3)	12 (17.1)
Other	1 (1.4)	2 (2.9)	0
GMFCS level, *n* (%)			
I	31 (44.9)	32 (46.4)	31 (44.3)
II	20 (29.0)	17 (24.6)	25 (35.7)
III	4 (5.8)	5 (7.2)	2 (2.9)
IV	14 (20.3)	15 (21.7)	12 (17.1)
Presence of epilepsy, *n* (%)	15 (21.7)	16 (23.2)	16 (22.9)
Previously treated with a BoNT‐A product, *n* (%)	45 (65.2)	47 (68.1)	46 (65.7)
MAS, mean (SD)			
PTMG	3.1 (0.3)	3.1 (0.3)	3.1 (0.5)
Elbow	2.7 (0.8)	2.9 (0.6)	2.7 (0.9)
Wrist	2.4 (0.9)	2.5 (1.1)	2.6 (1.2)

AboBoNT‐A, abobotulinumtoxinA; GMFCS, Gross Motor Function Classification System; BoNT‐A, botulinum neurotoxin A; MAS, Modified Ashworth scale; PTMG, primary targeted muscle group.

### Reduction of hypertonia/spasticity in the PTMG during cycle 1

**Figure 1:**
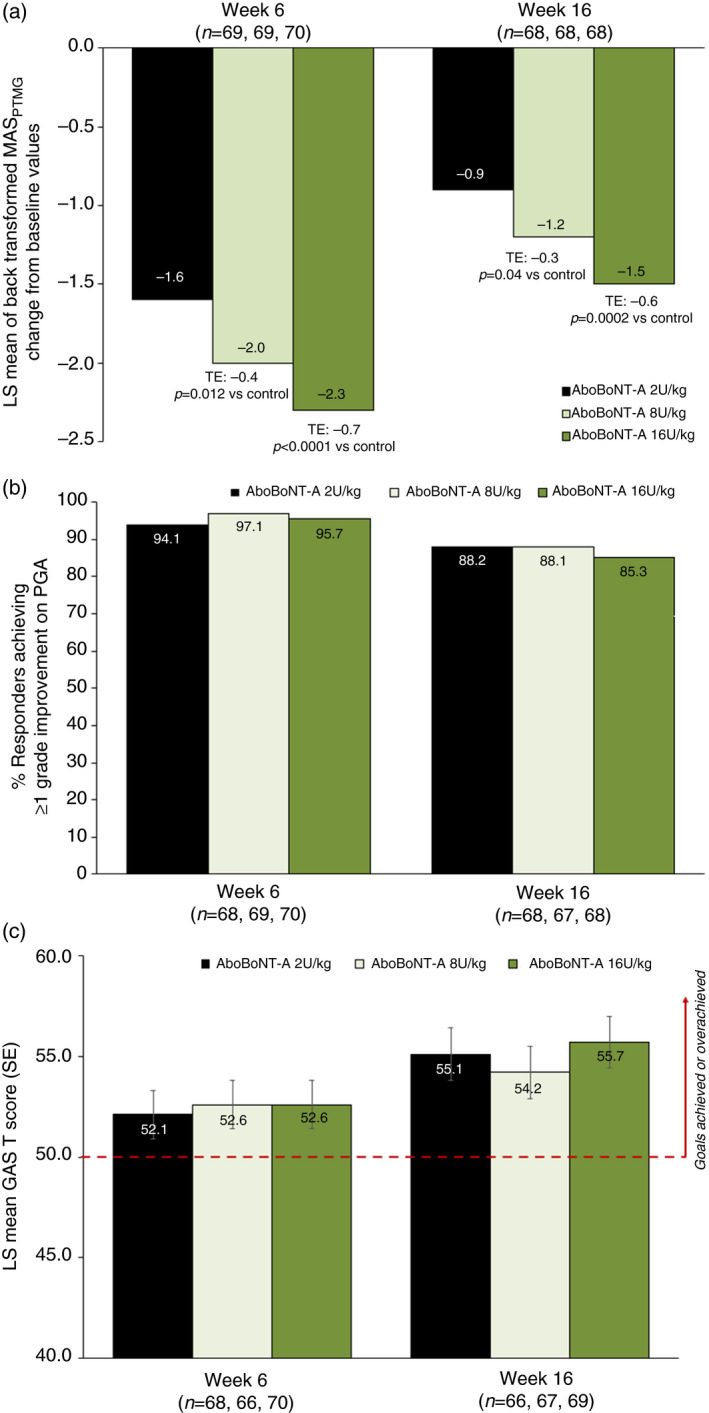
Treatment efficacy during cycle 1. (a) Least squares mean change in MAS_PTMG_from baseline to weeks 6 and 16. (b) Percentage of children achieving an improvement of at least one grade in PGA at weeks 6 and 16. (c) GAS T scores at weeks 6 and 16. AboBoNT‐A, abobotulinumtoxinA; GAS, goal attainment scaling; MAS, Modified Ashworth scale; LS, least squares; PGA, Physician Global Assessment; PTMG, primary targeted muscle group; SE, standard error; TE, treatment effect.

Mean changes from baseline to week 6 in the primary endpoint of MAS_PTMG_ were significant in both the 8U/kg and 16U/kg abobotulinumtoxinA dose groups versus the 2U/kg group; statistical superiority was maintained at week 16 (Table S3, online supporting information and Fig. 1a). Responder analyses confirmed that most children (81.2–94.3%) in each group had a clinically relevant (one grade) reduction in MAS_PTMG_ at week 6, and that most (61.8–83.8%) still had a relevant effect after 16 weeks (Fig. S2, online supporting information). Greater separation of groups was seen when the response definition was increased to at least two and at least three grades of MAS_PTMG_ improvement. MAS results by muscle group injected, irrespective of whether selected as PTMG (i.e. elbow, wrist, and finger flexors separately), are shown in Table S3.

Dose‐dependent changes in spasticity were also indicated by the results from the MTS. For PTMG elbow flexors, statistically significant improvements for both the 8U/kg and 16U/kg groups versus the 2U/kg group were seen at week 6 for the MTS angle of catch (X_V3_) and spasticity angle (X) (Table S4, online supporting information). Decreases from baseline were also observed in spasticity grade (Y) and were significant for the 16U/kg versus the 2U/kg group. For PTMG wrist flexors, the 16U/kg group (but not 8U/kg group) was significantly superior to the 2U/kg group at week 6 in X_V3_, X, and Y.

### Global and functional assessments during cycle 1

By week 6, all three study groups showed clinically relevant effects on PGA scores. The 8U/kg and 16U/kg abobotulinumtoxinA groups showed mean improvements of two grades, while the 2U/kg group showed an improvement of 1.8; there was no statistical difference between the groups (Fig. 1b, Table S3). These consistently positive results translated into very high responder rates across all three treatment groups as assessed by the clinician (Fig. 1b). Greater separation of groups was again seen when the response definition was increased to at least two and at least three grades of PGA improvement (Fig. S2).

GAS T scores at weeks 6 and 16 were consistently above 50.0 (indicating primary goal attainment at least as expected) for all three groups, with no significant difference between groups (Fig. 1c, Table S3). Most children across the three groups achieved their primary goals (70.6–75.8%) at week 6.

Children with hemiparesis in all three groups showed improvements from baseline in AHA scores at week 6 with no statistically significant differences observed (*n*≤31/group). All three treatment groups showed improvements in passive range of motion for the forearm pronators at week 6 with no statistically significant differences between groups. Only four children in each treatment group had a passive range of motion assessment in their shoulder muscles during cycle 1, and all showed improvement.

### Efficacy outcomes in cycles 2 to 4

The mean time from injection in cycle 1 to retreatment in cycle 2 was slightly longer in the 8U/kg and 16U/kg abobotulinumtoxinA groups compared with the 2U/kg group, and increased with dose (mean±SD of 23.9±10.5 and 25.6±10.3 vs 22.4±8.2 weeks respectively). For cycle 1, most children who received a second injection were retreated between 16 and 28 weeks; however, a substantial proportion of patients did not require reinjection until 34 weeks or later (18.8% in the 2U/kg group, 24.6% in the 8U/Kg group, and 24.3% in the 16U/kg group) (Table S5, online supporting information). The mean time to retreatment in subsequent treatment cycles was 19.4 weeks after the second injection and 17.4 weeks after the third injection (doses combined).

Improvements in MAS_PTMG_, MTS, PGA, and GAS T scores were similar between the 8U/kg and 16U/kg doses at week 6 across the subsequent treatment cycles, and the magnitude of improvement was generally consistent with cycle 1 (Table 2). MAS consistently improved by approximately two grades from baseline to week 6 of each treatment cycle, and the proportions of responders achieving clinically relevant (≥1) improvements in the MAS and PGA remained consistently high.

**Table 2:** Efficacy over cycles 2 to 4

	Cycle 2 (week 6)		Cycle 3 (week 6)		Cycle 4 (week 6)	
	8U/kg AboBoNT‐A	16U/kg AboBoNT‐A	8U/kg AboBoNT‐A	16U/kg AboBoNT‐A	8U/kg AboBoNT‐A	16U/kg AboBoNT‐A
MAS_PTMG_ ^a^	*n*=72	*n*=71	*n*=35	*n*=45	*n*=14	*n*=23
Mean (SD) at baseline of cycle	3.1 (0.3)	3.1 (0.5)	3.1 (0.4)	3.1 (0.6)	3.2 (0.4)	3.1 (0.3)
Mean (SD) change at week 6	–2.2 (1.0)	–2.3 (1.0)	–2.1 (1.1)	–2.0 (1.2)	–1.6 (1.3)	–1.8 (1.0)
*n* (%) children with ≥1 grade reduction	65/70 (92.9)	61/69 (88.4)	33/35 (94.3)	40/44 (90.9)	11/14 (78.6)	18/20 (90.0)
PGA score	*n*=86	*n*=90	*n*=45	*n*=57	*n*=20	*n*=33
Mean (SD) score at week 6	2.0 (1.0)	2.0 (1.1)	2.0 (1.2)	1.9 (1.1)	2.1 (0.9)	1.6 (1.1)
*n* (%) children achieving PGA score ≥1	80/85 (94.1)	83/89 (93.3)	40/44 (90.9)	48/56 (85.7)	20/20 (100.0)	25/31 (80.6)
GAS T score						
Mean (SD) score at week 6	51.9 (9.5)	53.4 (9.1)	49.1 (10.4)	48.7 (8.7)	43.7 (8.4)	48.1 (9.0)
*n* (%) children achieving primary goal (score ≥0)	64/84 (76.2)	72/87 (82.8)	24/45 (53.3)	39/55 (70.9)	6/20 (30.0)	21/31 (67.7)

^a^ Children who had the same PTMG throughout the study. AboBoNT‐A, abobotulinumtoxinA; MAS, Modified Ashworth scale; PTMG, primary targeted muscle group; PGA, Physician Global Assessment; GAS, goal attainment scaling.

### Safety and tolerability

In cycle 1, the proportion of children who had at least one TEAE reported was lower in the 16U/kg abobotulinumtoxinA group compared with the 8U/kg and 2U/kg groups (Table 3). The most frequently reported TEAEs (>5% of participants) in any treatment group were related to common childhood infections, the most common being upper respiratory tract infection (8.6% and 11.4% vs 7.1% of children in the 8U/kg and 16U/kg vs 2U/kg groups). Serious TEAEs were reported for 2.9% of children in the 8U/kg and 16U/kg groups versus 4.3% in the 2U/kg group; none were considered study‐related. Muscular weakness was the only treatment‐related TEAE reported in more than one child in any group (4.3% and 5.7% vs 1.4% of children in the 8U/kg and 16U/kg vs 2U/kg groups); these events were transient, mainly mild‐to‐moderate, and localized. One severe, non‐serious TEAE of muscular weakness (received 8U/kg) was considered treatment‐related. A second child (received 8U/kg) had mild generalized weakness that resolved without sequelae.

**Table 3:** Treatment‐emergent adverse events (TEAEs) in cycle 1

TEAE category, *n* (%)	2U/kg AboBoNT‐A (*n*=70)	8U/kg AboBoNT‐A (*n*=70)	16U/kg AboBoNT‐A (*n*=70)
Any TEAE	45 (64.3)	40 (57.1)	33 (47.1)
Intensity of TEAE			
Mild	38 (54.3)	34 (48.6)	25 (35.7)
Moderate	14 (20.0)	13 (18.6)	15 (21.4)
Severe	2 (2.9)	4 (5.7)	1 (1.4)
Any related TEAE	2 (2.9)	6 (8.6)	6 (8.6)
Any serious TEAE	3 (4.3)	2 (2.9)	2 (2.9)
Any TEAE leading to withdrawal	2 (2.9)	0	0
Any TEAE leading to death	0	0	0

AboBoNT‐A, abobotulinumtoxinA.

The incidence of treatment‐related TEAEs, including muscular weakness, was highest in cycle 1 and was generally reduced in the later cycles. Three (4.3%), no (0%), and one (2.2%) children in the 8U/kg group, and four (5.7%), five (5.6%), and one (1.8%) children in the 16U/kg group reported muscular weakness in cycles 1, 2, and 3 respectively; no event of muscular weakness was reported in cycle 4. Other treatment‐related TEAEs in cycles 2 to 4 (all *n*=1) included vomiting, seizure, fatigue, injection‐site pain, injection‐site rash, injection‐site bruising, hyperhidrosis, and arthralgia. No clinically meaningful changes attributable to abobotulinumtoxinA treatment were observed in the laboratory, electrocardiogram, or vital sign parameters during the study. Overall, 2.3% of children, all of whom had received prior treatment with BoNT‐A for spasticity, showed a seroconversion for neutralizing antibodies to BoNT‐A with no meaningful impact on safety and treatment efficacy.

## Discussion

In this phase 3, pivotal study, doses of 8U/kg and 16U/kg abobotulinumtoxinA met the primary endpoint of superiority compared with the 2U/kg low‐dose active control group with a dose‐dependent reduction from baseline in muscle tone as assessed by the MAS_PTMG_ during cycle 1. For a more comprehensive assessment approach, the study design also included global and functional outcomes. Under this multidimensional approach, and within the limitations of the study design (where children were randomized to a particular dose group, regardless of individual presentation), children in all three groups showed relevant improvements. Statistical differences versus the 2U/kg low‐dose control group were not shown for functional endpoints. Efficacy benefits were generally sustained over the 1‐year study with repeat treatment cycles of abobotulinumtoxinA plus home exercises, where doses of 8U/kg or 16U/kg abobotulinumtoxinA were given in the upper limb muscles. These doses (up to a total body dose of 30U/kg or a maximum of 1000U, when both upper and lower limbs were treated) were well tolerated.

This study was primarily designed for registration purposes and, as such, the primary efficacy objective was to show a reduction in upper limb spasticity. The MAS was chosen as the relevant primary outcome measure partly because it also acts as a bridge to existing level 1 data in other spasticity indications, and because it is well accepted by regulatory authorities. While there was a significant difference in MAS_PTMG_ between the 8U/kg and 16U/kg doses compared with the 2U/kg low‐dose control, it is important to note that the dose‐dependent efficacy of the abobotulinumtoxinA was most clearly differentiated when the threshold of the response definition was increased to at least two or three grades of MAS improvement. This may, in part, be explained by the mandatory instigation of the HETP, which may have enhanced the magnitude of reduction of muscle tone across all dose groups thus requiring a higher threshold for differentiation. The enforced use of injection guidance may also have improved treatment efficacy versus prior studies. Of note, the study design included MTS as a measure that is considered more consistent with the velocity‐dependent definition of spasticity.^18^ Dose‐dependent improvement in MTS assessments with greater superiority in reducing spasticity for the 16U/kg dose versus control support the primary efficacy findings. In general, there was greater separation of dose effect in the elbow flexors than the wrist flexors.

The degree to which the dose‐dependent reductions in spasticity seen on the MAS or MTS translate into functional benefits experienced by patients remains a controversial question in the field, and reviews of prior studies have only found conflicting evidence that treatment with BoNT‐A (with varying levels of occupational therapy) improves upper limb function or quality of life.^8^, ^20^, ^21^, ^22^ This is likely to be because studies such as ours randomly assign children to predefined dose groups, whereas it is well accepted that functional improvement after BoNT‐A treatment in clinical practice depends on the ability of clinicians to carefully choose doses dependent on several factors including the child’s underlying motor control and weakness. While dose‐dependency of effect was anticipated in terms of muscle overactivity, it was not at all expected for the functional measures because of this lack of ability to tailor dosing. In this study, all three groups showed relevant reductions in muscle tone and spasticity, and these were accompanied by highly relevant improvements in function‐related and global response measures. This included the relatively small subgroup of children with hemiparesis that was assessed with the AHA.^23^ GAS responder rates were generally higher at week 16, indicating a time lag from peak abobotulinumtoxinA effect on MAS to goal attainment, which may be expected as children learn to complete tasks with reduced spasticity.

Meta‐analyses have concluded that occupational therapy is effective in reducing spasticity and improving quality of movement, goal attainment, and overall performance,^5^, ^24^ and it is important to recognize that the HETP can be considered relatively intensive as compared with most standards of care. Moreover, the exercises chosen in the HETP were specifically chosen to support the chosen goals; although best efforts were made to standardize the minimum frequency and intensity of occupational therapy input, the contents of the home program varied according to individual needs. In this study, the clinical ‘pharmacodynamic’ measures of tone and spasticity were sensitive enough to demonstrate differential efficacy of the three abobotulinumtoxinA doses. However, in terms of overall function and goal attainment, and because of the study design, it is impossible to separate out the effects of therapy from those of abobotulinumtoxinA. On one hand, the HETP may have enhanced the reduction in muscle tone and spasticity across all treatment groups to such a degree that our ability to detect any dose‐dependent differences on functional outcomes was limited. On the other hand, it is possible that the results in the 2U/kg group reflect efficacy of low abobotulinumtoxinA doses associated with efficacy of the HETP. These possible explanations are not mutually exclusive and both may have contributed to our observations.

Adverse events reported in the study were mostly mild‐to‐moderate and included common childhood illnesses. Importantly, this study did not indicate a dose‐dependent increase in adverse events with increasing dose group. No child had a serious adverse event that was considered related to treatment, and no child discontinued because of a TEAE. However, one non‐serious, severe TEAE of muscular weakness with the 8U/kg dose was considered treatment‐related, and another child receiving 8U/kg had mild generalized weakness that did not re‐occur with subsequent treatment. Approximately half of the children had simultaneous injections into the upper and lower limbs during cycles 2 to 4, and there was no observable difference in the safety profile versus children only injected in the upper limb(s). Evaluation of safety by total body dose did not reveal any safety concerns with the higher total body dose groups up to the maximum dose (30U/kg or total 1000U). It has recently been suggested that studies in pediatric spasticity should assess potential muscle atrophy through pre‐ and post‐baseline measurements of muscle volumes and morphology using techniques such as serial magnetic resonance imaging.^25^ Most of the evidence to date is based on animal studies and population norm volunteers, and further research into the impact of myologic changes with repeat BoNT‐A treatment is warranted. In this study of up to four repeat injection cycles, while we did not look for potential atrophy, we did not see any evidence of a cumulative effect on adverse events, including muscle weakness.

In comparison with most previous studies of BoNT‐A for pediatric upper limb spasticity, this study has several important strengths including its size, duration, and inclusion of repeat cycles. Investigators were given the flexibility to retreat according to individual presentation rather than at fixed time points. We chose to assess the need for retreatment at 16 weeks instead of the standard 12‐week time point because accumulating evidence shows that the effects of abobotulinumtoxinA last longer than 12 weeks,^26^ especially in children.^27^ Another strength was the substantial training given: clinicians were trained and certified on the use of each outcome measure, and occupational therapists received significant training on how to implement the HETP for maximal compliance and in accordance with the child’s individual needs. Whether or not families can maintain this high level of compliance outside the study setting should be explored in more naturalistic studies.

A key limitation is the lack of a true placebo group and, as discussed above, the combined effects of the low 2U/kg dose with the intensive HETP may have influenced the results. Although it has recently been proposed that it is not feasible to conduct effective blinding in placebo‐controlled trials of BoNT‐A,^25^ the inclusion of a low‐dose control group mitigates this issue as some pharmacological effect is expected. However, as part of the informed consent, physicians and families were aware that all children would receive different doses of abobotulinumtoxinA, and expectations of an ‘active’ treatment may also have influenced outcomes. Although the multidimensional approach to assessment can be considered a strength of the study, another limitation is that we did not use the Manual Ability Classification System to describe our patients, whereas this and other functional measures would have been of interest. We did include AHA assessments for children with unilateral CP, but assessments were limited to those centers that had received certification. Finally, the study was not designed to compare between the 8U/kg and 16U/kg dose levels because, in practice, physicians require dosing flexibility to tailor treatment.

In summary, treatment with 8U/kg or 16U/kg abobotulinumtoxinA in the affected upper limb significantly reduced muscle tone and spasticity compared with the low‐dose 2U/kg control. Treatment with abobotulinumtoxinA plus HETP was associated with global improvement and high goal attainment, and therapeutic benefits were sustained with repeat treatment cycles where doses of 8U/kg or 16U/kg abobotulinumtoxinA were administered in the upper limb muscles. These data formed the basis of regulatory approval for abobotulinumtoxinA in several countries. Further studies in routine practice would help clarify the place of BoNT‐A in the long‐term management of spasticity, including how it is best combined with occupational therapy for functional improvement and other therapeutic approaches such as surgery.

## Conflict of interest

MRD, AT, JCR, ND, MB, RA, IM‐T, JO, and ED were investigators in Ipsen‐sponsored clinical trials, and they or their institutions received payment for participation. In addition, MRD reports personal fees from Ipsen, Allergan, and Kashiv Pharma for consultancy. AT reports research support and educational grants from Ipsen, and personal fees for consultancy from Ipsen. JCR reports personal fees for consultancy and speaking from Ipsen. ND reports research support from Ipsen, Allergan, and Merz, and personal fees for consultancy and speaking from Ipsen and Allergan. MB reports research support from Ipsen, Allergan, and Merz, and personal fees for consultancy and speaking from Ipsen and Allergan. RA and IM‐T have nothing further to report. JO reports consultancy fees for Ipsen and Allergan. ED reports personal fees from Ipsen and Allergan for speaking, Solstice Neurosciences for consultancy, and serves on a US speaker bureau. PP was employed by Ipsen at the time of study.

## Supporting information


**Appendix S1:** Institutional review boards.

**Table S1:** PGA

**Table S2:** Summary of goals selected at baseline cycle 1

**Table S3:** Cycle 1 efficacy endpoints

**Table S4:** MTS scores in cycle 1

**Table S5:** Time from injection to retreatment (cycle 1)

**Figure S1**: Study disposition.

**Figure S2:** Responder analyses at week 6 cycle 1 for MAS in the PTMG and PGA of treatment response.

## Data Availability

Where patient data can be anonymized, Ipsen will share all individual participant data that underlie the results reported in this article with qualified researchers who provide a valid research question. Study documents, such as the study protocol and clinical study report, are not always available. Proposals should be submitted to DataSharing@Ipsen.com and will be assessed by a scientific review board. Data are available beginning 6 months and ending 5 years after publication; after this time, only raw data may be available.
