# Supplementary material for: Efficacy and safety of abobotulinumtoxinA for upper limb spasticity in children with cerebral palsy: a randomized repeat‐treatment study
Source: Dev Med Child Neurol. 2020 Nov 18;63(5):592–600. doi: 10.1111/dmcn.14733 (PMC8048784; doi:10.1111/dmcn.14733)
Supplement: Supplementary file 2 — Table S1: PGA [file DMCN-63-592-s001.pdf]

Table S1. Physicians Global Assessment

Investigators were asked to *"rate the response to treatment in the subject's upper limb(s) since the start of the study"* on a 9 point rating scale.

-4	Markedly worse
-3	Much worse
-2	Worse
-1	Slightly worse
0	No change
+1	Slightly improved
+2	Improved
+3	Much improved
+4	Markedly improved.
