# Supplementary material for: Efficacy and safety of abobotulinumtoxinA for upper limb spasticity in children with cerebral palsy: a randomized repeat‐treatment study
Source: Dev Med Child Neurol. 2020 Nov 18;63(5):592–600. doi: 10.1111/dmcn.14733 (PMC8048784; doi:10.1111/dmcn.14733)
Supplement: Supplementary file 3 — Table S2: Summary of goals selected at baseline cycle 1 [file DMCN-63-592-s004.pdf]

Table S2. Summary of Goals Selected at baseline Cycle 1

The GAS is a functional scale used to measure progress towards individual therapy goals.[1] Goals should fulfil the principle of SMART – Specific, Measurable, Attainable, Measurable and Timely and are ranked according to their importance to the parent(s)/guardian(s)/child. After goal identification, the physician and/or therapist rates the level of difficulty of each chosen goal.

Once the goals have been set, the next step is to define 'expected' outcome of each goal and scale it into the 5-point scale below. The outcome required in order to reach each score of the scale has to be determined at baseline, and before any subsequent treatment

+2 = Much more than expected outcome

+1 = Somewhat more than expected outcome

0 = Expected outcome

-1 = Somewhat less than expected outcome

-2 = Much less than expected outcome

	**Control Group**	**Treatment Groups**	
**Goal Selected, n (%)**	**AboBoNT-A 2U/kg (*n*=69)**	**AboBoNT-A 8U/kg (*n*=69)**	**AboBoNT-A 16U/kg (*n*=70)**
**Primary Selected Goals**			
**Active Function**			
Involving Affected Arm More in Daily Activities	24 (34.8%)	16 (23.2%)	22 (31.4%)
Reaching	16 (23.2%)	16 (23.2%)	9 (12.9%)
Use of Limb as a Helping Hand to Stabilize	7 (10.1%)	5 (7.2%)	13 (18.6%)
Grasp and Release	5 (7.2%)	7 (10.1%)	5 (7.1%)
**Passive Function**			
Dressing	9 (13.0%)	5 (7.2%)	4 (5.7%)
Improve Range of Movement	2 (2.9%)	9 (13.0%)	4 (5.7%)
Donning/Tolerating Splints	0	3 (4.3%)	0
Overall Ease of Care	1 (1.4%)	1 (1.4%)	0
Hygiene	0	0	2 (2.9%)
**Pain**	1 (1.4%)	0	2 (2.9%)
**Other**	3 (4.3%)	7 (10.1%)	9 (12.9%)

[1] Turner-Stokes L. Goal attainment scaling (GAS) in rehabilitation: a practical guide. Clin Rehabil 2009; 23(4):362-70.

Individual Selected Goals (regardless of whether primary)			
**Active Function**			
Involving Affected Arm More in Daily Activities	31 (44.9%)	28 (40.6%)	31 (44.3%)
Reaching	21 (30.4%)	25 (36.2%)	26 (37.1%)
Use of Limb as a Helping Hand to Stabilize	17 (24.6%)	18 (26.1%)	18 (25.7%)
Grasp and Release	17 (24.6%)	21 (30.4%)	14 (20.0%)
**Passive Function**			
Dressing	12 (17.4%)	16 (23.2%)	7 (10.0%)
Improve Range of Movement	8 (11.6%)	16 (23.2%)	7 (10.0%)
Donning/Tolerating Splints	2 (2.9%)	4 (5.8%)	3 (4.3%)
Overall Ease of Care	3 (4.3%)	1 (1.4%)	1 (1.4%)
Hygiene	0	1 (1.4%)	4 (5.7%)
**Pain**	2 (2.9%)	1 (1.4%)	6 (8.6%)
**Other**	10 (14.5%)	11 (15.9%)	19 (27.1%)
