# Supplementary material for: Efficacy and safety of abobotulinumtoxinA for upper limb spasticity in children with cerebral palsy: a randomized repeat‐treatment study
Source: Dev Med Child Neurol. 2020 Nov 18;63(5):592–600. doi: 10.1111/dmcn.14733 (PMC8048784; doi:10.1111/dmcn.14733)
Supplement: Supplementary file 4 — Table S3: Cycle 1 efficacy endpoints [file DMCN-63-592-s008.pdf]

Table S3. Cycle 1 efficacy endpoints

	6 weeks			16 weeks		
	**AboBoNT-A 2U/kg (*n*=69)**	**AboBoNT-A 8U/kg (*n*=69)**	**AboBoNT-A 16U/kg (*n*=70)**	**AboBoNT-A 2U/kg (*n*=68)**	**AboBoNT-A 8U/kg (*n*=68)**	**AboBoNT-A 16U/kg (*n*=68)**
MAS_{PTMG}						
LS mean ± SEM [95% CI] of ranked change from baseline	125.8 ± 6.6 [112.7, 138.9]	102.5 ± 6.6 [89.5, 115.6]	85.4 ± 6.6 [72.3, 98.5]	123.5 ± 6.4 [110.8, 136.3]	105.4 ± 6.5 [92.6, 118.2]	89.9 ± 6.5 [77.0, 102.8]
Back transformed values in original scale; LS mean	-1.6	-2.0	-2.3	-0.9	-1.2	-1.5
Difference in LS Means		-0.4	-0.7		-0.3	-0.6
p value		*p*=0.012	*p*<0.0001		*p*=0.043	*p*=0.0002
n (%) children with ≥1-grade reduction	56 (81.2)	61 (88.4)	66 (94.3)	42 (61.8)	51 (75.0)	57 (83.8)
n (%) children with ≥2-grade reduction	32 (46.4)	47 (68.1)	55 (78.6)	22 (32.4)	26 (38.2)	35 (51.5)
n (%) children with ≥3-grade reduction	14 (20.3)	25 (36.2)	35 (50.0)	7 (10.3)	18 (26.5)	21 (30.9)
MAS_{elbow}	*n*=63	*n*=63	*n*=62	*n*=62	*n*=62	*n*=60
LS mean ± SEM [95% CI] of ranked change from baseline	115.1 ± 5.4 [104.4, 125.7]	86.4 ± 5.6 [75.2, 97.5]	78.4 ± 5.6 [67.4, 89.5]	111.6 ± 5.4 [101.0, 122.2]	90.3 ± 5.7 [79.1, 101.6]	81.3 ± 5.6 [70.1, 92.4]
Back transformed values in original scale; LS mean	-1.1	-1.7	-1.9	-0.6	-0.9	-1.1
Difference in LS Means		-0.6	-0.8		-0.3	-0.5
p value		*p*=0.0003	*p*<0.0001		*p*=0.007	*p*<0.0001

MAS_{wrist}	*n*=50	*n*=53	*n*=61	*n*=50	*n*=53	*n*=59
LS mean ± SEM [95% CI] of ranked change from baseline	90.1 ± 5.5 [79.3, 100.9]	81.3 ± 5.5 [70.4, 92.3]	74.8 ± 5.2 [64.5, 85.0]	86.2 ± 5.5 [75.4, 97.0]	85.7 ± 5.5 [74.8, 96.7]	76.4 ± 5.2 [66.0, 86.7]
Back transformed values in original scale; LS mean	-1.4	-1.6	-1.7	-0.9	-0.9	-1.1
Difference in LS Means		-0.2	-0.3		-0.0	-0.2
p value		ns.	*p*=0.030		ns.	ns.
MAS_{finger}	*n*=23	*n*=23	*n*=19	*n*=23	*n*=23	*n*=19
LS mean ± SEM [95% CI] of ranked change from baseline	47.5 ± 3.1 [41.3, 53.7]	33.2 ± 3.3 [26.6, 39.7]	35.1 ± 3.6 [27.8, 42.4]	42.2 ± 3.6 [34.9, 49.5]	37.1 ± 3.7 [29.6, 44.6]	31.7 ± 4.2 [23.2, 40.1]
Back transformed values in original scale; LS mean	-0.6	-1.5	-1.4	-0.8	-1.1	-1.4
Difference in LS Means		-0.8	-0.7		-0.3	-0.6
p value		*p*=0.0008	*p*=0.0066		ns.	ns.
PGA score						
LS mean ± SEM [95% CI] of ranked change from baseline	97.1 ± 7.1 [83.1, 111.0]	109.5 ± 7.0 [95.6, 123.4]	109.7 ± 7.1 [95.8, 123.7]	100.1 ± 6.7 [86.8, 113.3]	96.5 ± 6.8 [83.1, 109.9]	107.7 ± 6.8 [94.3, 121.1]
Back transformed values in original scale; LS mean	1.8	2.0	2.0	1.8	1.7	1.9
Difference in LS Means		0.2	0.2		-0.1	0.1
p value		ns.	ns.		ns.	ns.
n (%) children achieving PGA score ≥1	64 (94.1%)	67 (97.1%)	67 (95.7%)	60 (88.2%)	59 (88.1%)	58 (85.3%)

n (%) children achieving PGA score ≥2	43 (63.2%)	47 (68.1%)	52 (74.3%)	40 (58.8%)	38 (56.7%)	49 (72.1%)
n (%) children achieving PGA score ≥3	13 (19.1%)	18 (26.1%)	20 (28.6%)	16 (23.5%)	11 (16.4%)	21 (30.9%)
GAS T score						
LS mean ± SEM	52.1 ± 1.2	52.6 ± 1.2	52.6 ± 1.2	55.1 ± 1.3	54.2 ± 1.3	55.7 ± 1.3
Difference in LS Means [95% CI]		0.5 [-2.7, 3.7]	0.5 [-2.6, 3.7]		-0.9 [-4.4, 2.7]	0.6 [-2.9, 4.1]
n value		ns.	ns.		ns.	ns.
n (%) children achieving primary goal (score ≥0)	48 (70.6%)	50 (75.8%)	52 (74.3%)	57 (86.4%)	55 (82.1%)	56 (81.2%)
Passive range of motion for forearm pronators,	*n*=40	*n*=39	*n*=44	*n*=39	*n*=38	*n*=43
LS mean ± SEM change	1.5 ± 3.9	-2.4 ± 4.1	9.9 ± 3.8	2.8 (3.7)	4.5 ± 4.0	11.3 ± 3.6
Difference in LS mean vs. control [95% CI]		-3.9 [-14.2, 6.4]	8.4 [-1.4, 18.2]		1.8 [-7.8, 11.3]	8.6 [-0.5, 17.7]
n value		ns.	ns.		ns.	ns.
Passive range of motion for shoulder muscles*	*n*=4	*n*=4	*n*=4	*n*=4	*n*=4	*n*=4
Mean ± SD change (shoulder flexion)	2.5 ± 5.0	2.5 ± 8.7	5.0 ± 10.0	0.0 ± 8.2	6.3 ± 12.5	3.8 ± 12.5
Mean ± SD change (shoulder abduction)	0.0 ± 8.2	6.3 ± 12.5	3.8 ± 12.5	6.3 ± 9.5	0.0 ± 0.0	-1.3 ± 14.9
Assisting Hand Assessment (unilaterally affected children only)	*n*=24	*n*=28	*n*=31	Not assessed		
LS mean ± SEM change	4.6 ± 1.8	4.8 ± 1.6	3.7 ± 1.6			
Difference in LS mean vs. control [95% CI]		0.2 [-4.4, 4.8]	-0.9 [-5.4, 3.6]			
p value		ns.	ns.			

**No statistics performed due to small sample size.*
