# Supplementary material for: Efficacy and safety of abobotulinumtoxinA for upper limb spasticity in children with cerebral palsy: a randomized repeat‐treatment study
Source: Dev Med Child Neurol. 2020 Nov 18;63(5):592–600. doi: 10.1111/dmcn.14733 (PMC8048784; doi:10.1111/dmcn.14733)
Supplement: Supplementary file 5 — Table S4: MTS scores in cycle 1 [file DMCN-63-592-s003.pdf]

Table S4. Modified Tardieu Scale Scores in Cycle 1

	6 weeks			16 weeks		
	AboBoNT-A 2U/kg (*n*=69)	AboBoNT-A 8U/kg (*n*=69)	AboBoNT-A 16U/kg (*n*=70)	AboBoNT-A 2U/kg (*n*=68)	AboBoNT-A 8U/kg (*n*=68)	AboBoNT-A 16U/kg (*n*=68)
**Tardieu Scale score (PTMG = elbow)**						
Angle of arrest (X_{V1})						
LS mean ± SEM change	-1.2 ± 1.2	3.4 ± 1.2	2.2 ± 1.4	1.6 ± 1.0	1.0 ± 1.1	2.3 ± 1.3
Difference in LS mean vs control [95% CI]		4.5 [1.2, 7.9]	3.4 [-0.2, 7.0]		-0.6 [-3.5, 2.3]	0.7 [-2.4, 3.8]
p value vs. 2U/kg		*p*=0.0089	ns.		ns.	ns.
Angle of catch (X_{V3})						
LS mean ± SEM change	33.7 ± 5.4	52.5 ± 5.5	56.4 ± 6.4	16.6 ± 5.1	29.4 ± 5.2	42.1 ± 6.2
Difference in LS mean vs control [95% CI]		18.8 [3.4, 34.1]	22.7 [6.4, 39.0]		12.8 [-1.7, 27.3]	25.5 [10.1, 40.8]
p value vs. 2U/kg		*p*=0.0170	*p*=0.0067		ns.	*p*=0.0014
Spasticity angle (X)						
LS mean ± SEM change	-34.4 ± 5.4	-49.7 ± 5.5	-54.8 ± 6.4	-14.5 ± 5.0	-28.9 ± 5.2	-40.1 ± 6.2
Difference in LS mean vs control [95% CI]		-15.3 [-30.7, 0.0]	-20.4 [-36.7, -4.1]		-14.4 [-29.0, 0.1]	-25.6 [-41.0, -10.1]
p value vs. 2U/kg		*p*=0.0500	*p*=0.0144		ns.	*p*=0.0014
Spasticity grade (Y)						
LS mean ± SEM [95% CI] in ranked change	72.5 ± 4.4 [63.7, 81.3]	62.5 ± 4.5 [53.6, 71.4]	50.1 ± 5.2 [39.7, 60.5]	69.4 ± 4.0 [61.5, 77.3]	60.6 ± 4.1 [52.5, 68.7]	52.4 ± 4.8 [42.7, 62.0]
LS mean (back transformed)	-0.5	-0.8	-1.1	-0.1	-0.3	-0.5
Difference in LS means vs control		-0.3	-0.6		-0.2	-0.4
p value vs. 2U/kg		ns.	*p*=0.0013		ns.	*p*=0.0063

**Tardieu Scale score (PTMG = wrist)**						
Angle of arrest (X_{V1})						
LS mean ± SEM change	5.6 ± 2.8	-0.4 ± 2.7	7.4 ± 2.4	6.4 ± 3.1	-3.3 ± 3.0	4.6 ± 2.7
Difference in LS mean vs control [95% CI]		-6.0 [-12.9, 1.0]	1.8 [-4.6, 8.2]		-9.7 [-17.4, -1.9]	-1.8 [-8.9, 5.3]
p value vs. 2U/kg		*p*=0.0909	ns.		*p*=0.0155	ns.
Angle of catch (X_{V3})						
LS mean ± SEM change	54.1 ± 8.3	58.7 ± 8.1	87.9 ± 7.3	46.6 ± 9.6)	46.0 ± 9.4	58.7 ± 8.4
Difference in LS mean vs control [95% CI]		4.6 [-16.2, 25.4]	33.8 [14.9, 52.7]		-0.6 [-24.7, 23.5]	12.1 [-9.8, 34.1]
p value vs. 2U/kg		ns.	*p*=0.0007		ns.	ns.
Spasticity angle (X)						
LS mean ± SEM change	-51.1 ± 6.7	-59.5 ± 6.5	-79.9 ± 5.9	-42.2 ± 8.2	-48.7 ± 7.9	-53.9 ± 7.2
Difference in LS mean vs control [95% CI]		-8.4 [-25.1, 8.4]	-28.8 [-44.0, -13.6]		-6.4 [-27.0, 14.1]	-11.7 [-30.3, 7.0]
p value vs. 2U/kg		ns.	*p*=0.0003		ns.	ns.
Spasticity grade (Y)						
LS mean ± SEM [95% CI] in ranked change	49.2 ± 4.4 [40.3, 58.1]	43.9 ± 4.3 [35.3, 52.5]	35.1 ± 3.9 [27.2, 42.9]	46.7 ± 4.7 [37.4, 56.0]	47.0 ± 4.5 [38.0, 56.1]	37.6 ± 4.1 [29.4, 45.9]
LS mean (back transformed)	-0.4	-0.6	-0.8	-0.3	-0.3	-0.6
Difference in LS means vs control		-0.2	-0.4		0.0	-0.2
p value vs. 2U/kg		ns.	*p*=0.0072		ns.	ns.
