# Supplementary material for: Efficacy and safety of abobotulinumtoxinA for upper limb spasticity in children with cerebral palsy: a randomized repeat‐treatment study
Source: Dev Med Child Neurol. 2020 Nov 18;63(5):592–600. doi: 10.1111/dmcn.14733 (PMC8048784; doi:10.1111/dmcn.14733)
Supplement: Supplementary file 6 — Table S5: Time from injection to retreatment (cycle 1) [file DMCN-63-592-s007.pdf]

Table S5. Time from Injection to Retreatment (Cycle 1) (mITT Population)

	Control Group	Treatment Groups	
Visit	AboBoNT-A 2U/kg (*n*=69)	AboBoNT-A 8U/kg (*n*=69)	AboBoNT-A 16U/kg (*n*=70)
**Total subjects retreated**	**58 (84.1%)**	**60 (87. 0%)**	**60 (85.7%)**
Before Week 16	0	0	0
At Week 16	15 (21.7%)	19 (27.5%)	8 (11.4%)
At Week 22	23 (33.3%)	17 (24.6%)	23 (32.9%)
At Week 28	7 (10.1%)	7 (10.1%)	12 (17.1%)
At Week 34	7 (10.1%)	6 (8.7%)	3 (4.3%)
At Week 40	3 (4.3%)	5 (7.2%)	7 (10.0%)
At Week 46	3 (4.3%)	3 (4.3%)	2 (2.9%)
At Week 52	0	3 (4.3%)	5 (7.1%)
After Week 52	0	0	0

Eligibility for retreatment in the next treatment cycle was assessed at Week 16; if not eligible for retreatment they returned every 6±2 weeks until they required retreatment, or until Week 52. Up to 4 treatments could be administered in the study. Time from injection to retreatment (weeks) is calculated as: (retreatment date - injection date + 1) / 7.
