# Supplementary figures and images for: Efficacy and safety of abobotulinumtoxinA for upper limb spasticity in children with cerebral palsy: a randomized repeat‐treatment study
Source: Dev Med Child Neurol. 2020 Nov 18;63(5):592–600. doi: 10.1111/dmcn.14733 (PMC8048784; doi:10.1111/dmcn.14733)

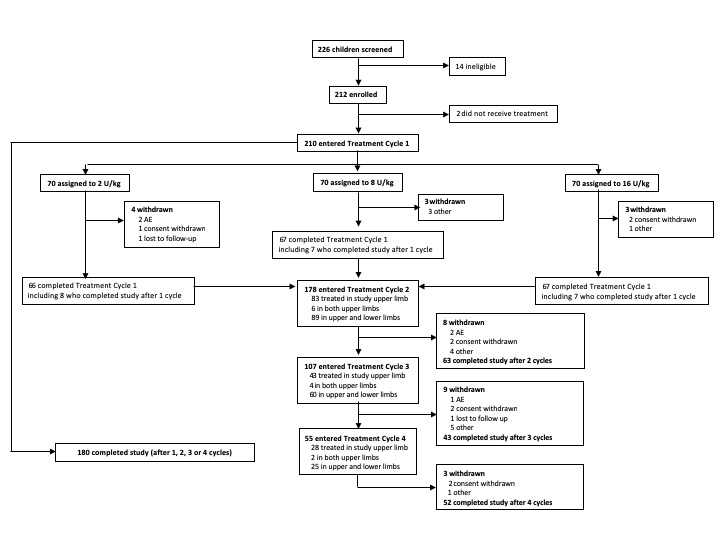

Supplement: Supplementary file 7 — Figure S1: Study disposition. [file DMCN-63-592-s002.jpeg]
