# Supplementary material for: Efficacy and safety of abobotulinumtoxinA for upper limb spasticity in children with cerebral palsy: a randomized repeat‐treatment study
Source: Dev Med Child Neurol. 2020 Nov 18;63(5):592–600. doi: 10.1111/dmcn.14733 (PMC8048784; doi:10.1111/dmcn.14733)

Figure S1. Responder analyses at Week 6 Cycle 1 for (a) Modified Ashworth Scale in the Primary Target Muscle Group (MAS_{PTMG}) (b) Physicians Global Assessment of treatment response (PGA)

(a) Responder analyses at Week 6 Cycle 1 for MAS_{PTMG}

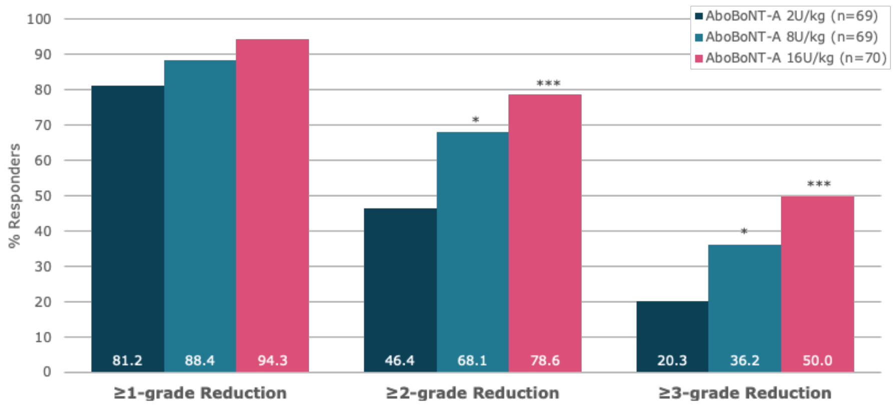

* p<0.05, ***p<0.001 vs. low dose control group

(b) Responder analyses at Week 6 Cycle 1 for PGA

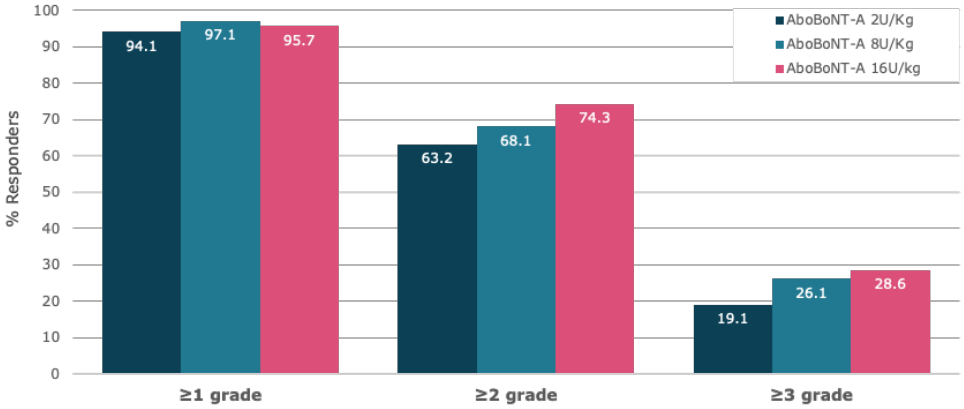

Supplement: Supplementary file 8 — Figure S2: Responder analyses at week 6 cycle 1 for MAS in the PTMG and PGA of treatment response. [file DMCN-63-592-s005.pdf]
